# Integrated multi-analytical screening approach for reliable radiocarbon dating of ancient mortars

**DOI:** 10.1038/s41598-022-07406-x

**Published:** 2022-02-28

**Authors:** Giulia Ricci, Michele Secco, Anna Addis, Anna Pistilli, Nereo Preto, Gian Pietro Brogiolo, Alexandra Chavarria Arnau, Fabio Marzaioli, Isabella Passariello, Filippo Terrasi, Gilberto Artioli

**Affiliations:** 1 Department of Geosciences, University of Padova, Via Giovanni Gradenigo 6, 35131 Padua, Italy; 2 Inter-Departmental Research Centre for the Study of Cement Materials and Hydraulic Binders (CIRCe), University of Padova, Via Giovanni Gradenigo 6, 35131 Padua, Italy; 3 Department of Cultural Heritage (DBC), University of Padova, Piazza Capitaniato 7, 35139 Padua, Italy; 4 Bruker Italia S.R.L. Daltonics Division, Via Cluentina 26/R, 62010 Macerata, Italy; 5 Centre for Isotopic Research on Cultural and Environmental Heritage (CIRCE), University of Campania “Luigi Vanvitelli”, Viale Carlo III 153, San Nicola La Strada, 81020 Caserta, Italy

**Keywords:** Chemistry, Materials science

## Abstract

Radiocarbon dating of the carbonate binder of historical mortars is a strategic research topic not lacking in complexities. The critical step is the separation of anthropogenic CaCO_3_-binder from other carbonate sources that could severely affect the resulting dates. Here we present a complete procedure for the processing and characterization of difficult mortars and of the separated binder fractions in order to assess a priori the chances of positively dating the mortar, and produce a binder fraction yielding the most reliable radiocarbon dates possible. Two complex architectural case studies from Northern Italy are presented and discussed in detail: the churches of Santa Maria Maggiore (Lomello, Pavia) and Santa Maria (Torba, Varese). The results support that both the reliability assessment and the successful radiocarbon dating are possible through a multi-analytical approach encompassing mineralogical and petrographic characterization, X-ray powder diffraction, scanning electron microscopy, measurement of carbon and oxygen stable isotopes, and optical cathodoluminescence.

## Introduction

Radiocarbon dating (^14^C) of archaeological and historical buildings is mostly based on dating organic materials. In the last 60 years, the possibility of using mortar as a material indicating the construction date of a building has been investigated, and there are examples of mortar dating studies that improved the chronological characterization of their investigated historical events^1,2^. Mortar-based materials are undoubtedly contemporary to the building and their methods of production make them valuable objects for radiocarbon dating a building’s time of construction^3,4^. ^14^C on mortars exploits the uptake of atmospheric CO_2_ by reaction with lime putty; atmospheric carbon is then stored into CaCO_3_-binder during the carbonation process. This process makes mortars suitable for radiocarbon dating, since ^14^C is fixed into the carbonate binder^5–7^.

Even if the method is conceptually simple, problems in the correct age estimation are common and are often related to the separation and selection of the pure binder fraction avoiding contaminants. The most common contaminants are: (i) geologic or fossil carbonate and lumps of original limestone, which affect the dating by incorporating dead carbon and severely overestimating the age. These components are generally defined as geogenic carbonate; (ii) secondary alteration processes, delayed hydraulic reactions and formation of long-term phases containing carbonate, which could be responsible of an underestimation of the age^8–11^. Large efforts were devoted by the scientists to find an efficient binder isolation method and control, but more dedicated studies in this peculiar field are still needed^12,13^.

In this framework, we present our approach in dating mortars which is based on the significance of a complete characterization of the studied samples. An appropriate chemical and mineralogical characterization of both mortar and isolated binder greatly enhances the understanding of the material’s features, allowing a preliminary assessment of the reliability of the binder for radiocarbon dating, and afterwards, the choice of the pre-treatments to be applied. Our approach includes, in brief: (i) a multi-analytical characterization of the material, in order to evaluate the crystal-chemical evolution of the sample and to identify potential dating contaminants; (ii) a careful binder extraction and processing in order to separate/eliminate the aggregate contaminants; (iii) a characterization of the extracted binder to check for the successful purification treatment; (iv) radiocarbon dating of the purified fraction.

In this research paper, mortar samples from two different archaeological sites in the north-western Italian region Lombardy are presented and discussed. Beside mortars, radiocarbon dating of lime lumps was also considered as may provide an alternative in radiocarbon dating, due to the carbonation process of calcium hydroxide contained in lime putty. In the same manner as the mortar matrix, the atmospheric CO_2_ is fixed in the lumps to form calcium carbonate^8,14–16^.

The aim is to evaluate the effectiveness of both the binder separation procedures and, in particular, the isotopic analyses (stable carbon and oxygen isotopes) as complementary method in evaluating the suitability of a given sample for radiocarbon dating. The characterization of the isolated binder fraction should be preliminary with respect to the radiocarbon dating analysis in order to ensure the complete removal of the contaminants. In this step, different complementary techniques were used since results obtained by one technique alone may be often controversial or partial.

In this research work, X-ray powder diffraction (XRPD), optical cathodoluminescence (OM-CL) and stable carbon and oxygen isotopes analysis (δ^13^C and δ^18^O) were used together for the first time as complementary techniques in order to predict and select reliable candidates for radiocarbon dating. Moreover, capability and limits of each methods are evaluated and discussed.

X-ray powder diffraction (XRPD) is a powerful technique able to detect mineralogical phases resulting from pozzolanic reactions (C–S–H, AFm, M-S–H), delayed hydraulic reactions and newly formed phases containing carbonate formed over a relatively long period (LDHs), that could be responsible of an underestimation of the age^17–19^. LDHs, as hydrotalcite-like minerals, are mixed hydroxides with lamellar structure in which M^3+^ cations partially substitute for M^2+^ cations and the positive charge is balanced by anions (often carbonate) and water molecules arranged in interlayers in alternation with the octahedral layers^20,21^. The flexible layered structure favours dynamic exchanges of carbonate derived from atmospheric CO_2_ even under ambient conditions^22–24^, introducing recent carbon as contaminant in radiocarbon dating^11,25^.

Optical-cathodoluminescence (OM-CL) exploits the luminescence properties of crystals when irradiated by an electron beam. Luminescence studies have long been used by geologists in order to investigate the provenance, minerogenesis, sediment source, diagenesis and cementation history of different kind of rocks and minerals^26,27^. In minerals, the luminescence response depends on intrinsic (lattice defects) and extrinsic (trace elements) point defects^28^. OM-CL has been one of the main screening methods for the identification of geogenic CaCO_3_ aggregates in mortars. Observing the luminescence of varying colours emitted by grains of CaCO_3_, it may be possible to discriminate geogenic carbonates (contaminants) from anthropogenic carbonates of binders. Most geogenic forms of CaCO_3_, e.g., limestone, exhibit orange-red luminescence caused by the occurrence of Mn^++^ in the calcite crystal lattice. Anthropogenic CaCO_3_ shows instead dull luminescence, due to the Mn^++^/Fe^++^ ratio in the calcite related to the changing of Eh and pH conditions in the setting mortars^8,29–33^. When using cathodoluminescence to identify the absence of geogenic carbonates, there is an implicit assumption of geogenic carbonates being luminescent. This assumption is often justifiable, as limestones (generally used in mortar mixing) are almost always luminescent due to the Mn^++^ incorporation to the crystal structure^30,31^.

Finally, stable isotope analysis was used to test the nature of the carbonate phase due to the isotope fractionation of δ^13^C and δ^18^O isotopes during portlandite carbonation^34–40^. The stable carbon isotopes were exploited in order to differentiate the anthropogenic calcite versus any contamination. The ideal δ^13^C value of a binder carbonate, formed by the atmospheric CO_2_ absorption from calcium hydroxide, is between −27 and −20‰ VPDB^41^. In fact, variations from the ideal δ^13^C to more positive or negative values may indicate the presence of contaminants (geogenic carbonate, layered phases containing recent atmospheric CO_2_, etc.) that could invalidate the radiocarbon dating. The method has been extensively tested here, so that a detailed discussion of its applicability is provided.

### δ^13^C and δ^18^O isotopes analysis

The stable isotope fractionation of carbon and oxygen in mortars depends on the manufacture and hardening processes, especially on the isotopic content of the atmospheric CO_2_, the water present during the carbonation reaction, and the quality and quantity of the aggregates^42^.

The CaCO_3_-binder produced by carbonation process is identical in chemical composition to calcite produced at ambient temperature, however, the stable isotopic composition of anthropogenic as well as biogenic calcites may preserve information of the environment and conditions under which they were formed^41,43^.

According to the literature and experimental data^35–37,44–46^, the carbonate from lime mortar and/or cement and concrete forms under non-isotopic equilibrium conditions. The strong alkaline environment (pH > 11) and the consequent rapid absorption of CO_2_ directly from the atmosphere induce the formation of calcite with an extreme isotopic composition^36,44^. In strong alkaline conditions it is estimated that 2/3 of δ^18^O derives from the CO_2_ and 1/3 from OH^−^^41,47^. The isotope values are expressed relative to the VPDB-standard (Vienna Pee Dee Belemnite)^48^.

Ideally, δ^13^C and δ^18^O of a lime-based binder formed by the carbonation reaction thorugh direct absorption of atmospheric CO_2_, in a strong alkaline environment (pH > 11), are δ^13^C_matrix_ = −25‰ (or −20.7‰ according to^42^) and δ^18^O_matrix_ = −20‰VPDB. According to^41,49,50^, the ideal stable isotopes values of calcite formed directly by absorption of atmospheric CO_2_ are: δ^13^C_calcite_ between −27 and −20‰, and δ^18^O_calcite_ = −19‰. Such values best confirm that the CO_2_ incorporated in the carbonate derives from the atmosphere^41,51^ and therefore the isotopic ratios of the binder fraction are highly indicative of the purity of the binder, i.e., its anthropogenic nature^4,37,41^. Marzaioli et al.^4^, measured a similar range for δ^13^C of synthetic mortars (from −14.9 to −21.9‰) by calcination of a natural carbonate (δ^13^C = 2.9‰) and successive carbonation with natural air of the laboratory (δ^13^C = −11‰).

Indeed, variations from the ideal values of δ^13^C and δ^18^O towards positive or negative values indicate the presence of contaminants (such as geological carbonate, or newly formed phases that absorbed recent atmospheric CO_2_) that can affect the ^14^C dating results. In most cases, the calcite matrix can be easily distinguished from limestone aggregates (e.g., marine limestone, dolomite, marble etc.) by their isotope values. Therefore, stable isotope analysis is potentially a powerful sample screening method for ^14^C dating.

In Supplementary Figure S1 (modified from^36^), the ideal binder values (IBV) and the variations of the isotopic ratios related to the calcite formation conditions are reported. The variations of the isotopic values according to lines 1–4 indicate the different types of alteration mechanisms potentially present in the sample. Line 1 indicates a binder geologically contaminated by the presence of limestone (line 1a and 1b are deviations due to relicts of limestone used for burning or contamination by limestone aggregates); line 2 indicates variability in water or rainwater, as heavy sources and/or evaporation effect; line 3 indicates that organic carbon contaminants may be present in the binder, fed carbon to the primary CO_2_, or it may derive from biogenic alteration of anthropogenic calcite; line 4 indicates a variation due to either a primary source of CO_2_ with light oxygen, or exchange with water derived from a light-oxygen source.

### Archaeological contexts and mortar samples

The mortars investigated in the present work (see Table 1) were sampled from two different Lombard churches: Santa Maria Maggiore in Lomello (province of Pavia, coordinates 45.122594, 8.793882) and Santa Maria in the Abbazia di Torba complex (province of Varese, coordinates 45.729522, 8.863244).

**Table 1:** Mortar samples collected from the Lomello and Torba sites. The reported dates of the construction phases are those inferred from archaeological and historical information.

Site	Sample code	Sampling area	Construction phase
LOMELLO	LOM_1	Baptistery of Saint Giovanni ad Fontes, basis of the baptismal font	V-VI sec. AD
	LOM_2	Baptistery of Saint Giovanni ad Fontes, second baptismal font	VII-VIII sec. AD
	LOM_3	Church of Santa Maria Maggiore, South-west wall of the basement	V-VI century A.D. (oldest phase of the church) or X-XI?
	LOM_4	Church of Santa Maria Maggiore, South-west wall of the basement	V-VI century A.D. (oldest phase of the church) or X-XI?
	LOM_5	crypt, above the column	X–XI sec. AD (?)
	LOM_6	crypt, above the pulvinus of the column at the entrance	X–XI sec. AD (?)
	LOM_7	crypt, joint between the capital and the pulvinus of the same column as LOM-6	X–XI sec. AD (?)
TORBA (Castel Seprio)	TOR_1	Church, inside the bell tower	Before or after the crypt?
	TOR_2	Church, masonry in the corner	Before or after the crypt?
	TOR_3	Crypt, plaster on the lower part	I crypt–VII–VIII sec. AD
	TOR_4	Crypt, plaster on the upper part	I crypt–VII–VIII sec. AD
	TOR_5	Crypt, reconstruction of the apse, lower part	Later than I–X–XI sec. AD
	TOR_6	Crypt, reconstruction of the apse	Later than I–X–XI sec. AD
	TOR_7	Crypt, upper part, external perimeter area of the church	II crypt–VII–VIII sec. AD
	TOR_9	Buttress	III crypt
	TOR_10	Crypt	Later than II–VII–VIII sec. AD
	TOR_12	Building IV	Settlement IX–X sec. AD
	TOR_13	Roman walls	V sec. AD
	TOR_14	Tower	IX–X sec. AD
	TOR_15	Wall behind the church	Late mediaeval period
	TOR_16	Wall behind the church	Late mediaeval period

The sampling was carried out considering the sampling depth in order to account the delayed carbonation problem, as discussed by Lindroos et al.^52^, hence, were collected and analysed unaltered samples coming from the surface and not from deeper parts.

The sites were singled out for their historical and archaeological importance, despite of their very complex architectural developments. The mortars were carefully selected during archaeological excavations, on the account of their significance in the reconstruction and interpretation of the architectural sequence. The nature and features of the mortars proved to be a challenge for dating methods^38^ and actually stimulated the present investigation in order to develop more efficient purification protocols and reliability tests, such as the δ^13^C analysis used to verify the purity-grade of the extracted fine binder fractions.

**Figure 1:**
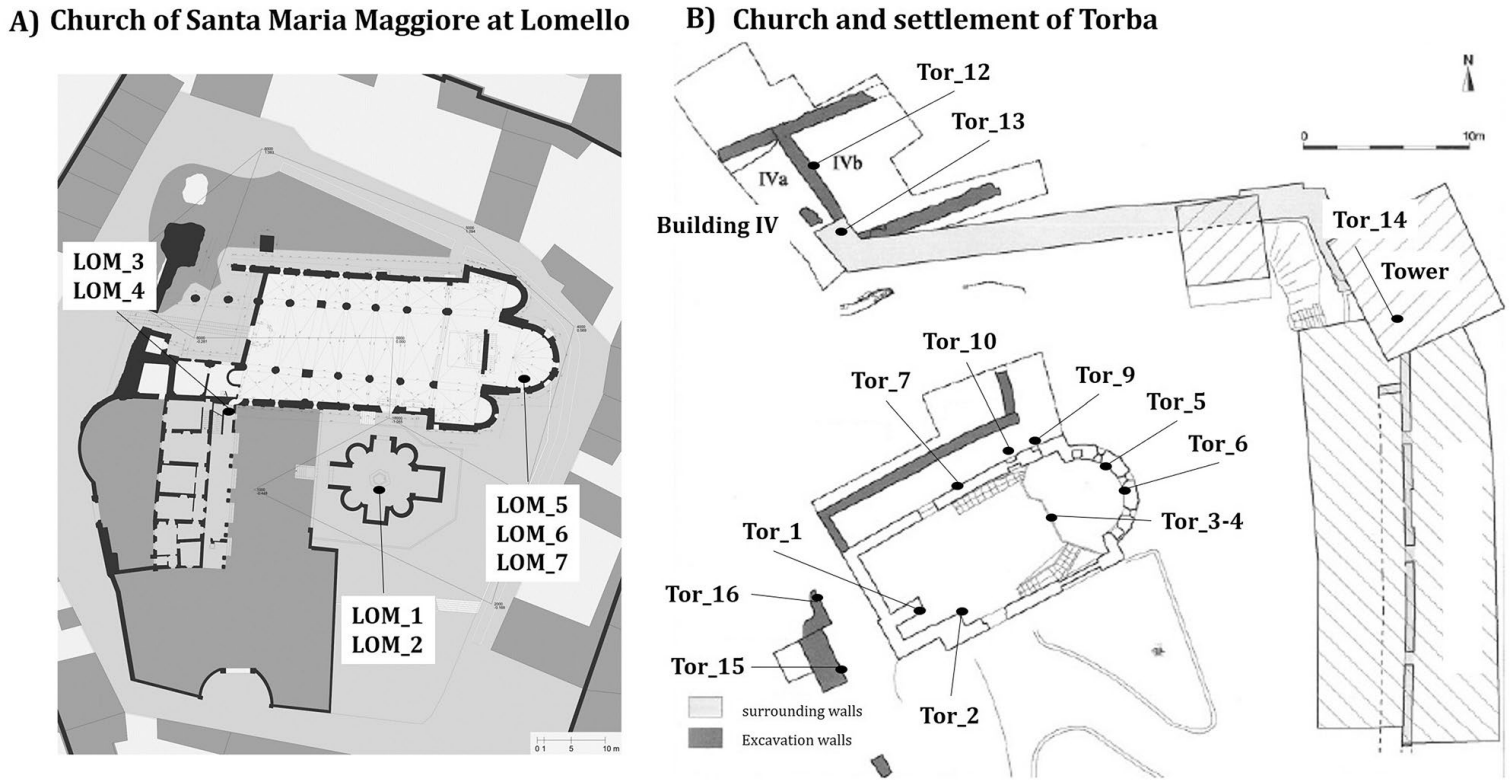
Maps of the two archaeological sites with indication of the collected mortar samples. (GIMP 2.10.10. GNU Image Manipulation Program, https://www.gimp.org/). (**A**) The church of Santa Maria Maggiore at Lomello (Pavia) and (**B**) the church of Santa Maria and the settlement of Torba complex (Castelseprio, Varese).

The church of Santa Maria Maggiore in Lomello, reconstructed in the year 1025 AD, is one of the most representative architectonic examples of First Romanesque art^53–55^. The church is part of a complex with an older baptistery, San Giovanni ad Fontes, built between the V and VIII century^56,57^. The mortar sampling was carried out into the baptistery (LOM_1 and LOM_2), southern wall of the church (LOM_3 and LOM_4) and the crypt (LOM_5, LOM_6 and LOM_7), as shown in Fig. 1A. Lime lumps (P) were also collected from three mortar samples (LOM_2, LOM_3 and LOM_4), characterized and dated by ^14^C.

The Torba site was funded as a military structure at the end of the IV or beginning of the V century, as a part of the Castel Seprio late roman fortification, and it was later (possibly from the VIII century) reused as a part of a Benedictine monastery consisting in a tower with a funerary floor and chapel, monastic spaces, a house (named building IV) hosting the non-religious members of the community (called *conversi*), and the church of Santa Maria. Recent analysis have defined four periods of evolution of the church (A, B, C, D), not totally clear^58,59^. During an excavation conducted in 2013–2014, a total of 14 mortar fragments were selected (Fig. 1B) and sampled from: the crypt of the church (TOR_1-7, TOR_9-10); the *so-called* Building IV, NW of the church (TOR_12); the Roman wall (TOR_13); the second floor of the tower NE of the church (TOR_14); the wall SW behind the church (TOR_15 and TOR_16). In addition, three lime lumps (P) manually collected from three mortar samples (TOR_7_P, TOR_10_P and TOR_12_P) were tested and radiocarbon dated.

## Results

### Mortars characterization

**Figure 2:**
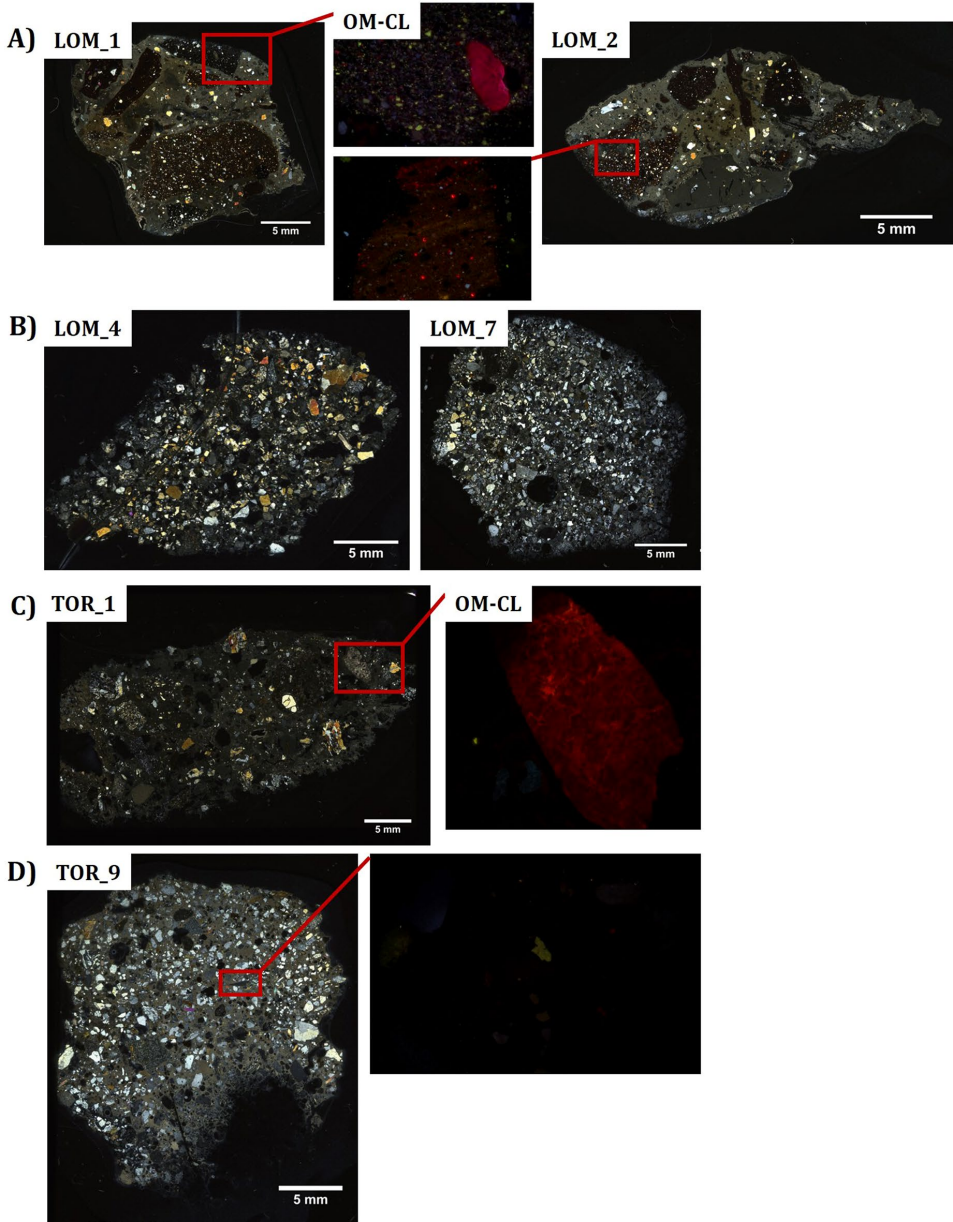
Representative mortar samples from Lomello and Torba under optical microscope in transmitted light. (**A**) polarized light micrographs of LOM_1 and LOM_2 thin sections (crossed polars), and OM-CL photos of the highlighted red squares; (**B**) polarized light micrographs of LOM_4 and LOM_7 thin sections (crossed polars). Polarized light micrographs of TOR_1 (**C**) and TOR_9 (**D**) thin sections (crossed polars), and OM-CL photos of the highlighted red squares.

The samples from the baptismal baths (LOM_1 and LOM_2) in the Baptistery of Saint Giovanni at Lomello, are macroscopically characterized by the presence of coarse-grained ceramic fragments used as aggregates. Under the optical microscope (Fig. 2), the matrixes present calcite interference colours, indicating the occurrence of carbonated lime binder. The large fragments of ceramic used as aggregates embed different types of inclusions, such as quartz and feldspars. Furthermore, limestone inclusions with the typical red/orange luminescence were observed by OM-CL (Fig. 2A), which may represent a potential problem in ^14^C dating because they could produce radiocarbon ages older than the mortars. The XRPD analysis (see Supplementary Table S1) of these two samples indicates that the mortars are basically composed by c.a. 30%wt of calcite, ascribable to both the binder and the limestone inclusions, 30%wt of quartz and feldspars, related to the aggregates, and c.a. 35%wt of clay minerals and amorphous phases, related to the ceramic aggregates. The collected mortars from the church at Lomello (LOM_3-7) are characterized by a calcic binder and silicate-based rock aggregates, such as quartzites, schists, serpentinites and amphibolites. The mortars of the crypt (LOM_5-7) are undoubtedly related to the foundational structures of the church, the binder/aggregate ratio is very low and mineralogical characterization carried out by XRPD on the bulk samples shows low amount of calcite between 4 and 13%wt (Supplementary Table S1).

The 14 mortar samples collected from the archaeological site of Torba are characterized by homogenous structural and compositional features, poor cohesion and by the occurrence of coarse aggregates. Microscopically (Fig. 2C and D), the binder matrixes present a microcrystalline texture of carbonate composition, sometimes associated with clay fractions dispersed homogeneously in the matrix. Quartz, feldspars, amphiboles, micas (muscovite and biotite), flint and fragments of metamorphic rocks such as schists and quartzites are present as aggregates. Lime lumps were identified in all samples by both OM and SEM observations. Three of them were manually isolated and collected from the bulk samples TOR_7, TOR_10 and TOR_12, and analysed by XRPD, OM-CL, isotope ratios and finally they were radiocarbon dated. Under OM-CL, carbonate aggregates are identified as luminescent centres in the matrixes, which generally show low-medium luminescence and therefore are prone to efficient separation of the binder.

**Figure 3:**
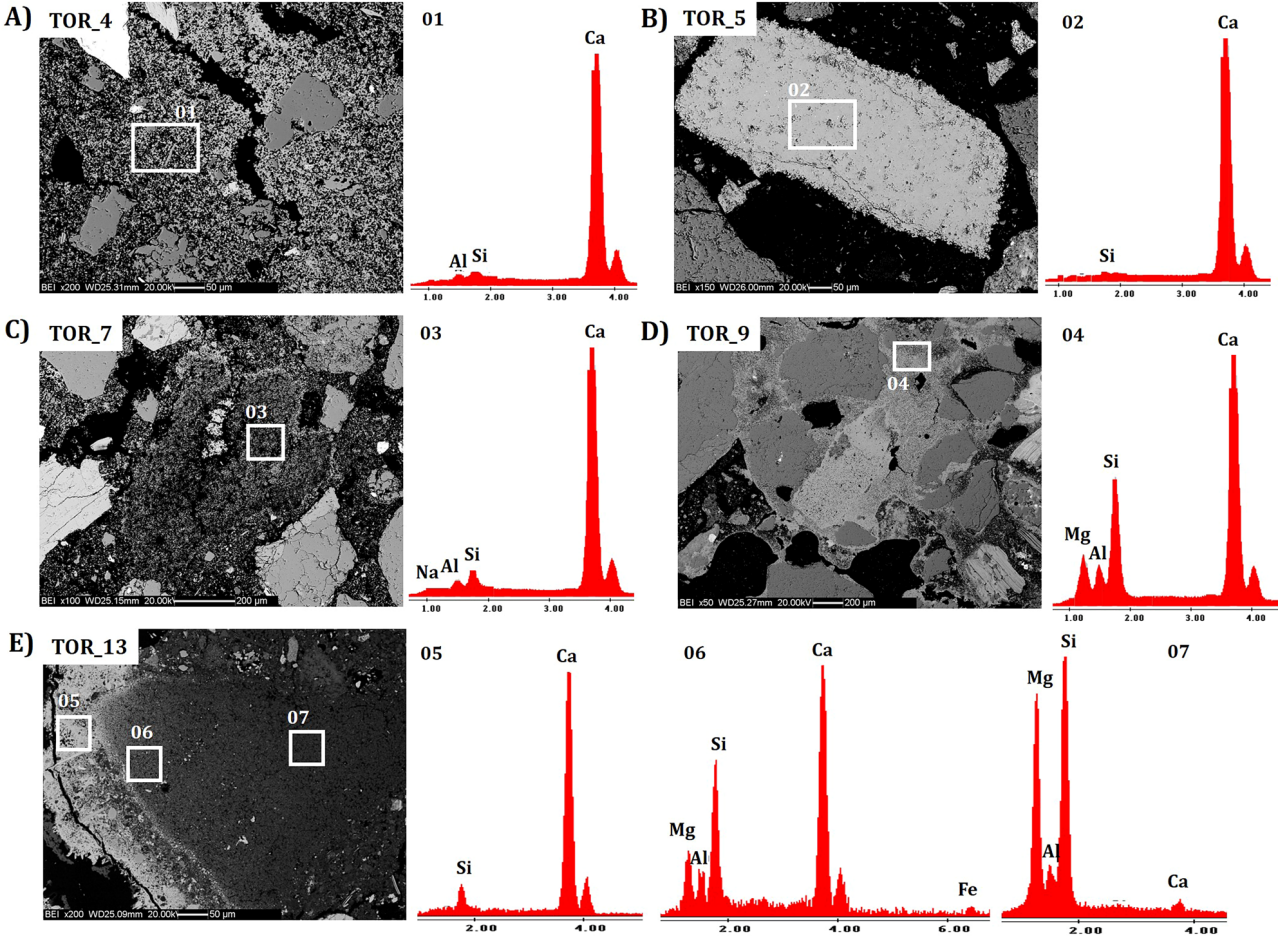
SEM–EDS microphotographs and elemental analyses of characteristic samples from Torba. (**A**) TOR_4, EDS microanalysis of the carbonate binding matrix (01); (**B**) image and microanalysis of a lump (02) within TOR_5; (**C**) TOR_7, microanalysis of the binding matrix (03); (**D**) TOR_9, microanalysis of the binding matrix (04); (**E**) TOR_13 microanalyses of a hydraulic lump (06, 07) and its rim (05).

Mineralogical investigation of the bulk samples, carried out by XRPD (Supplementary Table S1), shows the presence of quartz, albite, microcline, muscovite and amphiboles ascribable to the rock aggregates of the mortar, as seen by OM observations. This composition may be related to the use of local sand^60^. The occurrence of phyllosilicates (as chlorite) may be related to a silty fraction added to the lime mixture, probably related to an inaccurate purification of the aggregate prior to mortar mixing^61^. The presence of calcite (up to 33 wt%) is attributed to both the aerial reaction of the binder fraction and the presence of carbonate aggregates as seen by optical microscopy. Substantial content of amorphous phases (between 7 to 28 wt%) may be related to the occurrence in the binding matrices of paracrystalline phases, possibly related to long term products of pozzolanic-type reactions. Indeed, the XRPD analysis highlights the occurrence in few samples (TOR_2, TOR_9, TOR_14) of double layered hydroxides (LDH). The presence of these hydrotalcite-type compounds may be due to the interaction between lime and reactive silicate aggregates such as Mg-rich phyllosilicates (chlorite)^11,24,25^. Generally, the mortar samples from Torba present a mineralogical composition ascribable to aerial mortars obtained by a lime binder and silicate sand. However, a few samples such as TOR_10, TOR_12, TOR_13 and TOR_15, are characterized by a mixture mainly composed of sand and clayey soil, with the addition of a small quantity of lime, as they are characterized by low content of calcite and higher amounts of amorphous, chlorite and silicate phases. The amorphous content may indicate the presence of paracrystalline phases related to hydraulic reaction products such as C-S–H, AFm and M-S–H phases^19^. SEM–EDS results are consistent with those obtained by XRPD. The matrixes present microcrystalline texture, evidences of calcic lumps and, in almost all the samples, a homogenous composition mostly composed by Ca (Fig. 3A and B). TOR_7, TOR_9 and TOR_16 mainly present matrixes with carbonate composition associated with portions characterized by significantly higher Si, Al and Mg concentrations, whereas the lumps’ microanalyses suggest the use of a calcic binder (Fig. 3C and D). TOR_13 shows features indicative of a partial carbonation, heterogenous matrix mostly composed by Ca, Si, Al and Mg with lumps characterized by similar composition and microstructure (Fig. 3E). Generally, the presence of Si, Al and Mg in the binder matrixes may indicate the formation of hydrated magnesium silico-aluminate phases (M-A-S–H) after reaction between the lime binder and Mg-rich phyllosilicates (or other reactive silicates) of the aggregate fraction^19,62,63^. This is confirmed by the exceptional evidence of Mg-Si-Al-rich lumps in TOR_13, indicating pozzolanic reactions and the unusual formation of almost pure M-A-S–H lumps (Fig. 3E).

### Characterization of the binder fractions (SG) and lumps (P)

Representative mortar samples collected from both Lomello and Torba were subject to the separation procedure in order to separate the binder fraction (SG) from the contaminants and aggregates. The SGs were then characterized by XRPD, OM-CL and stable isotope analyses. The characterization of the separated binder fractions allows checking whether the samples are suitable for dating, to limit the number of samples to be dated and the relative costs, and to preliminary assess the possible causes of error.

**Figure 4:**
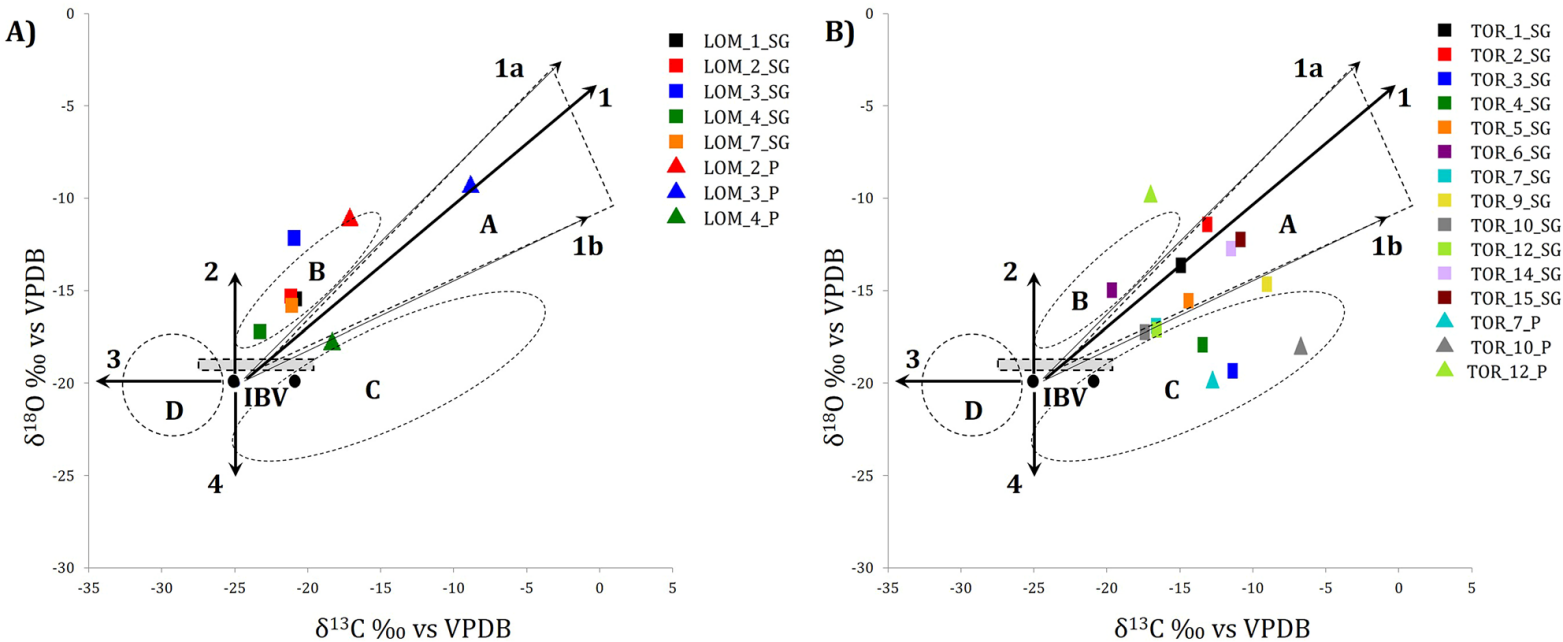
Isotopic ratios (δ^13^C and δ^18^O) diagram of both mortar binders (SG) and lumps (P) of Lomello (**A**) and Torba (**B**). The IBV points (dark circles) and bars (gray dashed rectangle) represent the Ideal Binder Values. Trend lines (1–4) and marked areas (**A**–**D**) as reported in Supplementary Figure S1 and discussed in the introduction. Sample TOR_13_SG is not inserted in the diagram because its low carbon content yields unreliable results.

Among the 7 mortar samples from Lomello, 5 samples were selected (LOM_1-4 and LOM_7) and subject to the purification procedure. As previously discussed, in these mortars the binder/aggregate ratio is very low, and consequently, the purification procedure was carried out very carefully to avoid loss of the scarce binder material. The LOM_SGs characterization indeed suggests that these SG fractions can reliably be used to date the construction time of the building. The δ^13^C values are between −23.6 and −21.1‰ (Table 2 and Fig. 4A), suggesting carbonate formation directly by absorption of atmospheric CO_2_^35,41^. The δ^18^O values are between −17.3‰ (sample LOM_4_SG) and −12.1‰ (sample LOM_3_SG). As discussed in literature, the enrichment of heavier oxygen isotopes with respect to a typical anthropogenic mortar carbonate may depend on the primary water source and/or to the evaporation of water during the hardening process of the mortars. Furthermore, it has been observed that this enrichment may also be due to re-equilibration with the silicate minerals, especially in cases of low binder/aggregate ratio^41,50,64,65^. However, as shown in Fig. 4A, the selected mortar samples (SGs) lay in the area B mostly indicating a small contamination ascribable to the oxygen fractionation of altered calcite or the use of isotopically heavy water^36,41,50^. The XRPD results (Table 2) show a mineralogical composition almost entirely constituted of calcium carbonate, and no evidences of geological carbonate contaminations in CL observations are detected. The presence of aragonite (metastable polymorph of calcium carbonate) in some samples (both from Lomello and Torba) is ascribable to the carbonation process^66^, since no evidence of shell fragments or other biogenic carbonates was observed during the characterization analyses. Therefore, these samples may be good candidates for the radiocarbon dating.

**Table 2:** Summary table of the characterization carried on the binder fractions and lumps of the selected samples. Mineralogical phases (by XRPD), stable isotopes, and luminescence (by OM-CL) characterization results of the binder separate (SG) and lump (P) samples of the Lomello and Torba mortars are presented.

Sample code	Fraction	Mineral phases	δ^13^C (‰)	δ^18^O (‰)	Luminescence
LOM_1	Binder (SG)	++Cc, --LDH, --Qtz	−21.1	−15.5	0
LOM_2		++Cc, -Clay	−21.4	−15.3	0
LOM_3		+Cc, ++Arg, --LDH	−21.2	−12.1	0
LOM_4		++Cc, -Clay, -Arg, --Qtz	−23.6	−17.3	0
LOM_7		++Cc, -Arg, (t)Dol	−21.4	−15.8	0
LOM_2	Lump (P)	++Cc, -Qtz	−17.3	−11.2	3
LOM_3		++Cc, -Arg, (t)Qtz	−9.0	−9.4	3
LOM_4		++Cc, -Qtz	−18.5	−17.9	3
TOR_1	Binder (SG)	++Cc, -Dol, --Clay, --Qtz	−15.1	−13.6	3
TOR_2		+Cc, +Dol, -Clay, -LDH, --Qtz	−13.3	−11.4	3
TOR_3		++Cc, -LDH, --Qtz	−11.6	−19.4	0
TOR_4		++Cc, --Qtz	−13.6	−17.9	0
TOR_5		Cc, -Clay, --Arg, (t) LDH	−14.6	−15.6	3
TOR_6		++Cc, -Qtz	−19.8	−15.0	0
TOR_7		++Cc, --Qtz, (t)Clay	−16.7	−16.9	0
TOR_9		++Cc, --Qtz, (t) LDH	−9.2	−14.7	1
TOR_10		++Cc, --Qtz, (t) LDH	−17.5	−17.3	0
TOR_12		++Cc, --Qtz, --Arg	−16.7	−13.1	0
TOR_13		Clay, M-A-S-H, --Cc	−12.2*	−21.9*	0
TOR_14		++Cc, -Clay, --LDH, --Qtz	−11.7	−12.7	2
TOR_15		+Cc, +Clay, +LDH, M-A-S-H	−11.0	−12.2	1
TOR_7	Lump (P)	++Cc	−12.9	−20.0	2
TOR_10		++Cc	−6.9	−18.1	0
TOR_12		++Cc, -Arg	−17.1	−9.8	0

Cc = calcite; Dol = dolomite; LDH = layer double hydroxides; Qtz = quartz; Arg = aragonite; M-A-S–H = hydrated magnesium-silicoaluminate phases (more details are provided in the supplementary material). (++) very abundant, (+) abundant, (-) scarce, (--) very scarce, (t) trace.

0 = dull, 1 = low, 2 = low-medium, 3 = medium-bright, 4 = bright. *very low signal of carbon.

On the other hand, the collected lime lumps (LOM_2-4_Ps) are characterized by: a mineralogical composition mostly of calcite, more positive δ^13^C values between −18.5‰ and −9.0‰ (Table 2) and a bright red luminescence (Supplementary Figure S3A). These features are indicative of the presence of limestone residues which were incompletely calcined^63^. In particular, sample LOM_3_P show a significant shift to heavier isotopic values, likely related to the isotopic signature of the limestone used for burning. LOM_2_P and LOM_4_P exhibit a milder shift towards heavier δ^13^C isotope values, likely due to small impurities of isotopically heavy limestone, since luminescence investigation on powdered lumps show a medium-bright luminescence. Therefore, the Lomello lime lumps (Ps) may not be good candidates for radiocarbon analyses due to the systematic presence of geological carbonate contaminations.

Concerning the case study of Torba, almost all the mortar samples were selected for the separation and characterization procedures. The extracted binder fractions show different features. Most of them show a dull luminescence, proving the efficacy of the separation procedure; however, a higher δ^13^C with respect to the ideal value IBV between −27 and −20‰ (VPDB) is observed, indicating some alteration and/or contamination of the mortar samples as the presence of other carbon sources, such as modern atmospheric carbon dioxide, secondary calcite and/or geological limestone^36,41,42^. SGs are mainly characterized by a mineralogical composition with high quantities of calcite and limited fractions of quartz, phyllosilicates (clays) and traces of LDHs. In TOR_1_SG and TOR_2_SG, dolomite is also present, probably due to the fine fraction of the dolomitic aggregate. Dolomite is also present in the bulk XRPD characterization. TOR_13_SG and TOR_15_SG show different mineralogical composition with less calcite, clays and presence of LDH phases. Furthermore, as suggested by SEM–EDS analyses, the mineralogical profiles observed by XRPD reported in Supplementary Figure S2 in the supplementary materials, are similar and attributable to those of M-S-H and M-A-S-H phases characterized by small particle size, low crystallinity and broad peaks of low diffracted intensity^67,68^.

The samples TOR_1_SG, TOR_2_SG, TOR_5_SG and TOR_14_SG show diffuse luminescent centres and, in general, a medium-bright luminescence intensity, probably due to residues of carbonate aggregates. The stable isotopic composition of these 4 samples, −15.1 < δ^13^C < −11.7‰ and −15.6 < δ^18^O < −11.4‰ (Fig. 4B, Table 2), indicate that these mortars are affected by limestone contamination. TOR_6_SG, TOR_7_SG, TOR_10_SG and TOR_12_SG have δ^13^C values between −19.8 and −16.7‰, dull luminescence, and mineralogical composition mostly of calcite, therefore these samples should be nearly free of contaminants.

TOR_3_SG and TOR_4_SG, with δ^13^C equal to −11.6‰ and −13.6‰ respectively, dull luminescence and the presence of LDH phases (in the case of TOR_3_SG), exhibit minimal contamination from atmospheric CO_2_ and recent CO_2_ likely absorbed by carbonate-containing double layered hydroxides (LDHs)^25,41^ and secondary phases.

The last two mortar samples, TOR_9_SG and TOR_15_SG, may be affected by different contaminations. The characterization shows dull luminescence and more positive isotopic values than the ideal ones. Furthermore, the XRPD results show the presence of calcite, low amount of quartz, clayey phases, LDHs and poorly crystalline phases such as M-A-S–H, particularly in TOR_15_SG. The enrichment of δ^13^C may be related to slow continuous calcite formation and segregation of ^12^C in the gas phase, and to the presence of isotopically heavy limestone impurities. Both calcite types may indeed have dull luminescence. High δ^13^C isotope values may be expected in mortar mixes composed by small amounts of lime binder and large quantities of silicates prone to delayed pozzolanic reactions, as in the case of sample TOR_15^41,65^.

Among the lime lump samples, TOR_7_P and TOR_10_P are characterized by white colour, mineralogical composition mainly composed of calcite, high δ^13^C and δ^18^O values, and dull luminescence. As in the case of the TOR_3_SG and TOR_4_SG, these lumps may present contamination due to precipitation of calcite formed from more recent atmospheric CO_2_. It is possible that also TOR_7_P and TOR_10_P contain secondary calcite formed sometime after the main carbonation reaction of the binder mortars, thus causing an underestimation of the measured radiocarbon date^14,69^. TOR_12_P is characterized by an essentially carbonate composition, and no geogenic-related cathodoluminescence signal is observed. Its δ^13^C value (−17.1‰) is close to the ideal values and the δ^18^O value (−9.8‰) suggests a heavy isotopic composition of the water interacting with the binder during the carbonation process, or alteration after the hardening process of the mortar^50^. However, TOR_12_P may be considered as a reliable lump sample for radiocarbon dating.

### Radiocarbon dating

The characterization carried on the separated binder fractions (SGs) and lumps (Ps) allowed to identify the suitable samples in order to date the construction phases of the archaeological sites. The choice of the samples for radiocarbon dating was made considering the archaeological relevance and the results obtained by XRPD, OM-CL and isotopic ratio of stable carbon isotopes δ^13^C characterization. Furthermore, for the sake of testing our method, radiocarbon dating of predicted unreliable samples was also performed.

In the following table (Table 3), ^14^C results including radiocarbon ages and calibrated calendar ages of all the selected and analysed samples are presented.

**Table 3:** Radiocarbon dating results.

CIRCE Code	Sample code	Area	Fraction	^14^C age (BP)	Cal. age range (1σ)	Cal. age range (2σ)
DSH6797	LOM_1	Baptistery	Binder SG	1525 ± 35	AD 444–600	AD 433–635
DSH6989	LOM_2			1356 ± 37	AD 645–759	AD 605–775
DSH6798	LOM_3	Church of Santa Maria Maggiore		1072 ± 27	AD 900–1020	AD 893–1025
DSH6990	LOM_4			877 ± 38	AD 1054–1221	AD 1043–1260
DSH6799	LOM_7	Crypt		1059 ± 28	AD 977–1024	AD 895–1030
DSH6781	LOM_2	Baptistery	Lump P	1731 ± 38	AD 253–383	AD 245–408
DSH6782	LOM_3	Church of Santa Maria Maggiore		18,181 ± 89	BC 20,296–20,096	BC 20,397–19,993
DSH6783	LOM_4			1415 ± 47	AD 603–656	AD 557–757
DSH6771	TOR_4	Crypt	Binder SG	1393 ± 33	AD 609–662	AD 596–675
DSH6773	TOR_7	Church walls		1417 ± 35	AD 605–653	AD 582–664
DSH6775	TOR_10			1275 ± 31	AD 680–770	AD 663–824
DSH5617	TOR_15	West wall behind the church		353 ± 30	AD 1478–1626	AD 1459–1635
DSH6774	TOR_7	Church wall	Lump P	1078 ± 28	AD 902–1017	AD 893–1024
DSH6777	TOR_10			988 ± 27	AD 1021–1147	AD 994–1155
DSH6768	TOR_12	Building IV		1110 ± 32	AD 895–989	AD 776–1018

The characterized mortar samples from Lomello (LOM_1-4_SGs and LOM_7_SG) were selected as good candidates for radiocarbon dating on the basis of their characterization, whereas the three lumps (LOM_2-4_Ps) present contaminations probably due to geological carbonates, detected by both stable isotopes and OM-CL characterization. All samples were nonetheless dated and the calibrated calendar ages were compared with those expected and discussed.

The two SG samples of the mortars collected from the Baptistery in Lomello (LOM_1-2_SG) show calibrated dates (AD 433–635 and AD 605–775, respectively) in agreement with the ages expected from historical and archaeological considerations (V-VI sec. AD and VII-VIII sec. AD, respectively). On the other hand, the calibrated age of the lump sample of the second bath (LOM_2_P, AD 245–408) is older than the mortar LOM_2 and consequently older than the expected date (VII-VIII sec. AD), as predicted by the characterization analyses showing that the sample is clearly contaminated by geological carbonate. Similar results are obtained for the samples related to the church and crypt of the Santa Maria Maggiore, where the SGs (LOM_3-4_SG and LOM_7_SG) show calibrated calendar ages (see Table 3) very close to the documented construction period of the church (X and XI century AD). Sample LOM_3_P, characterized by more positive δ^13^C value and bright red luminescence, and sample LOM_4_P, with medium-bright luminescence, show unreliable old ages of c.a. 20,000 B.C. and c.a. 600 A.D, respectively, demonstrating that these samples are not reliable candidates for radiocarbon dating due to contamination of geological carbonates, according to experimental characterization.

Prioritizing good candidates for radiocarbon dating and their relevance for archaeological questions, TOR_4_SG, TOR_7_SG and TOR_10_SG were the selected samples of the crypt (TOR_4) and of the outer wall of the Santa Maria church of Torba (TOR_7 and TOR_10). The sample TOR_15_SG, from the west wall behind the church, was also selected for the testing procedure: it is characterized by the presence of LDHs, high δ^13^C and δ^18^O values and dull luminescence, indicating the presence of contaminants which should bias its radiocarbon age. The three lump samples were also dated.

Radiocarbon dating results (Table 3) of the TOR_7_SG and TOR_10_SG, selected as reliable samples for radiocarbon measurements, show calibrated calendar dates between 582 and 824 AD, which are correct according to archaeological hypotheses^58^, where the assumption is that the constructions are older than the X century. Sample TOR_4_SG is radiocarbon dated to between 596 and 675 AD. Archaeological records temporally place the sample TOR_7 before TOR_10, while samples TOR_4 and TOR_7 belonging to the VII-VIII sec AD. The experimentally measured dates of TOR_4 and TOR_7 are essentially coeval, confirming the archaeological expectations. TOR_4_SG seemed to be slightly contaminated by recent carbon, as suggested by the isotope ratio measurements (δ^13^C = −13.6). However, XRPD and CL investigations did not detect any LDH phases and/or re-precipitated calcium carbonate. Another possibility in having recent carbon contaminants can be a mortar affected by a delayed carbonation process^52^. TOR_4 was collected from the basement of the church, and, as well as the other samples, the sampling was made considering the general problems related to delayed hardening in the sampling depth^52^.

The calibrated calendar age obtained for the sample TOR_15_SG (AD 1459 ‒ 1635) is too young. Archaeological studies predicted a late dating of this particular wall, however, the multi-analytical characterization allowed to identify different kind of contaminations in the sample that can lead to various uncertainties on the dating obtained. Stable isotopes results approach the isotopic composition of modern CO_2_ (δ^13^C between −9 and −6‰ (VPDB)^36^) suggesting the presence of contamination probably due to the LDH phases, detected by XRPD investigation, which incorporated CO_2_ after the hardening process^11,25^.

The calibrated age of lump sample TOR_12_P is correct (AD 776‒1018), in accordance with the indications of its mineralogical and isotopic characterization. On the other hand, the dates obtained for lump samples TOR_10_P and TOR_7_P are sensibly younger than those of the SGs of the same samples, and are to be considered unreliable, as already suggested by isotopic analyses. The general indication is that lime lumps must be very carefully controlled before radiocarbon dating.

## Discussion and conclusions

The multi-analytical approach used for characterizing the binder fraction of mortars in two historical sites of Lombardy, i.e. Lomello and Torba, is a promising protocol for a pre-selection of suitable samples for radiocarbon dating. The novel application of the chosen techniques on extracted binder fractions show how their complementarity can be effective overcoming the limits of each single technique.

The radiocarbon ages of the SG samples selected by multi-analytical characterization are consistent with ages expected from archaeological, historical, and textual information. Lime lumps have been shown to frequently include under-burnt limestone cores that seriously affect the radiocarbon ages, as observed by CL-OM and stable isotopes results. The use of lime lumps in dating must therefore be exerted with caution.

From an archaeological point of view, the VII century date for Santa Maria di Torba introduces a new chronology for this type of simple hall crypt surmounted by vaults: it would precede those with a western corridor, built around the middle of the VIII century in Pavia (as Santa Maria alle Cacce and San Salvatore/San Felice) and in the territory of Brescia (San Salvatore di Sirmione and San Giorgio di Montichiari), until now considered the oldest in northern Italy^59^.

In the church of Santa Maria di Lomello, stratigraphic analyses have identified the successive stages of the Romanesque construction. The dating of the mortars to around the year 1000 (compatible with the 1020 ± 72 thermoluminescence dating of a brick in the pilaster strip of the north perimeter, as reported in^70^) suggests that the church was built by Cunberto, the count of Lomello from 996, and his son Ottone, count of the palace and of Pavia from 999 to 1014.

The investigated case studies clearly demonstrate the importance of the mortar binder characterization by multiple techniques (isotopic signature, XRPD, and cathodoluminescence) to evaluate in detail the presence of possible contaminants and common biases affecting the radiocarbon measurements (Table 4).

**Table 4:** Summary table. The expected and the calibrated calendar ages of the Lomello and Torba samples are reported and compared.

Sample code	Area	Construction phase/expected age	Fraction	Radiocarbon contaminants?	^14^C Cal. AD age range (2σ)	Reliable calibrated calendar age?
LOM_1	Baptistery	V-VI sec. AD	SG	No	AD 433–635	Yes
LOM_2	Baptistery	VII-VIII sec. AD	SG	No	AD 605–775	Yes
			P	Yes, geogenic carbonate	AD 245–408	No (older)
LOM_3	Church of Santa Maria Maggiore	V-VI century A.D. (oldest phase of the church) or X-XI? (?)	SG	No	AD 893–1025	Yes
			P	Yes, geogenic carbonate	BC 20,397–19,993	No (older)
LOM_4	Church of Santa Maria Maggiore	V-VI century A.D. (oldest phase of the church) or X-XI? (?)	SG	No	AD 1043–1260	Yes
			P	Yes, geogenic carbonate	AD 557–757	No (older)
LOM_7	Crypt	X-XI? (?)	SG	No	AD 895–1030	Yes
TOR_4	Crypt, plaster on the upper part	I crypt–VII–VIII	SG	No (?)*	AD 596–675	Yes (?)*
TOR_7	Crypt, external perimeter area of the church	II crypt–VII–VIII	SG	No	AD 582–664	Yes
			P	Yes, recent CO_2_	AD 893–1024	No (younger)
TOR_10	Church walls, crypt	Later than II–VII–VIII	SG	No	AD 663–824	Yes
			P	Yes, recent CO_2_	AD 994–1155	No (younger)
TOR_12	Building IV	IX–X sec. AD	P	No	AD 776–1018	Yes
TOR_15	West wall behind the church	Late medieval period	SG	Yes, geogenic carbonate and recent CO_2_	AD 1459–1635	No (?)

*Isotope ratio measurements suggested TOR_4_SG to be slightly contaminated by recent carbon, however, XRPD and CL investigations did not detect any LDH phases and/or re-precipitated calcium carbonate. The date can be assumed to be correct.

Stable isotope analyses proved to be an effective tool to predict the unsuitability of a sample for ^14^C dating, effectively recording contaminations. However, it has to be noted that the use of isotopic data alone would have led to exclude various samples due to an isotope ratio not exactly coincident with the range of the ideal binder, as suggested by the data reported in the literature^36^ and reported in the supplementary materials of this article (Supplementary Figure S1).

The complementary techniques, such as XRPD and CL-OM used in the characterization procedures, fundamentally support the effectiveness of the separation procedure and considerably increase the chances of reliable dating. Integrating the obtained results, reliable samples can be chosen and radiocarbon dated. The XRPD analysis provides information on the mineralogical composition of the binder fraction identifying radiocarbon contaminants as LDHs, nevertheless, when calcite is identified as the major component of the binder fraction, this technique is not able to distinguish among geogenic, anthropogenic or secondary calcite. For this purpose, CL is generally applied in assessing the nature of the calcium carbonate, and the luminescence response caused by both geogenic and secondary calcite can be discussed with the isotopic values in order to better identify if it was an old or a young contamination.

It is proposed that the described protocol strengthen the whole procedure of radiocarbon mortar dating, based on solid experimental information.

## Materials and methods

### Analytical approach and methods

The adopted analytical approach consists of the following strategy: (i) chemical–mineralogical characterization of the mortars; (ii) multi-step purification procedure of the mortar binders; (iii) characterization of the extracted purified binder fractions and evaluation of the reliability of the selected samples for radiocarbon dating; (iv) graphitization and radiocarbon dating of the purified fractions.

Besides mortar samples, selected lime lumps manually collected from the same mortar samples were characterized and radiocarbon dated.

Characterization and purification procedures were performed at the CIRCe Centre in Padua (Department of Geosciences, University of Padova, Italy), whereas graphitization and AMS measurements were carried out at CIRCE Centre in Caserta (Department of Mathematics and Physics, University of Campania, Italy).

### Chemical and mineralogical characterization

The selected mortars were characterized by a multi-analytical approach aiming at assessing the nature of the binders and the presence of potential contaminants. Petrographic analyses were performed using a Nikon Eclipse ME600 optical microscope equipped with a Canon EOS 600D Digital camera on 30 μm thin-sections under parallel and crossed polars. Selected thin sections were observed under an optical cathodoluminescence microscope (OM-CL) in order to evaluate the presence of geogenic carbonates, using a petrographic microscope NIKON Labophot2-POL equipped with a cold cathode stage Cambridge Image Technology Ltd, CL8200 MK3 operated at a voltage of 15 kV and a current of 200 µA. Furthermore, thin sections covered with an ultrathin coating of graphite, were microstructurally and microchemically characterized through a CamScan MX2500 Scanning Electron Microscope (SEM) equipped with a LaB_6_ electron source and an EDS used to collect elemental microanalyses (system resolution of 126.8 eV for 5.9 eV Mn) through the SEMQuant Phizaf software, giving valuable information on the mineral phases, binder composition and presence of hydraulic reactions. Mineralogical quantitative phase analyses (QPAs) were performed by XRPD on fine powders obtained from bulk samples by micronization. XRPD analyses were performed using a Malvern PANalytical X’Pert PRO diffractometer in Bragg–Brentano geometry, Co–Kα radiation, 40 kV and 40 mA, equipped with a real-time multiple strip (RTMS) detector (X’Celerator by Malvern Panalytical). Data acquisition was performed by operating a continuous scan in the range 3°–85° 2θ, with a virtual step scan of 0.02° 2θ. Diffraction patterns were interpreted with X’Pert HighScore Plus 3.0 software by Malvern PANalytical, reconstructing mineral profiles of the compounds by comparison with ICDD and ICSD diffraction databases. QPAs were performed using the Rietveld method^71^ and refinements were accomplished using the TOPAS software (version 4.1) by Bruker AXS. The determination of both crystalline and amorphous content was calculated by means of the internal standard method with the addition of 20 wt% of zincite (ZnO) to the powders^72^.

### Multi-step purification procedure

The purification procedure of selected mortars was carried out in order to remove aggregates and potential dating contaminants by wet gravimetric sedimentation. The procedure consists in a sonication and wet gravimetric sedimentation in ultra-pure decarbonated water for 24 h, centrifugation and filtration of the fine fraction (labelled SG)^6,25,38,73^. The separation procedure needs between 15–30 g of mortars, and generally 10 to 100 mg of the fine fraction can be obtained depending on the ratio binder/aggregate.

### Characterization of the extracted fine binder and lime lumps

The ultra-pure fine binder fractions (SGs) and the lime lumps (Ps) were characterized before radiocarbon dating in order to verify the absence of contaminants. The characterization included: XRPD, OM-CL and stable carbon and oxygen isotope analyses. The latter was carried on by a Thermo Scientific Delta V Advantage Isotope Ratio Mass Spectrometer. In details, about 0.6 mg of SGs were weighted in exetainer vials. CO_2_ was developed at 70 °C by complete reaction with > 99% H_3_PO_4_ in a Gasbench II device connected to the spectrometer. Results were calibrated with two internal standards (sieved Carrara marble and Millipore Suprapur® carbonate), which are in turn periodically calibrated against the international reference carbonates NBS 19; NBS 18 and L-SVEC. A control standard (sieved Monzoni marble) was also run and treated equally to the samples and reproduced with external errors of better than 0.1‰ (1σ) for both carbon and oxygen. XRPD and OM-CL were applied to the separated binder fractions by adopting the same analytical protocols described above used for the bulk samples.

### Radiocarbon dating of the purified fractions and lumps

The SGs and Ps were digested under vacuum by means of a complete orthophosphoric acid attack for 2 h at 80°C^4^. The released CO_2_ was reduced to graphite on iron powder catalyst according to the CIRCE sealed tube reaction protocol^74^. In details, IAEA C1 historical series (mass of carbon vs apparent age) were used for background correction and IAEA C2 was used for normalization purposes^4^. ^14^C isotopic ratios were measured according to^75^ and corrected for fractionation and blank, normalised and R.C. ages were estimated (M and H 1977) and calibrated to absolute ages by means of OxCal 4.4.4^76^ and INTCAL20 calibration curve.

## Supplementary Information

Supplementary Information.

## Data Availability

All data are available in the main text or the supplementary materials.
