# Supplementary material for: Integrated multi-analytical screening approach for reliable radiocarbon dating of ancient mortars
Source: Sci Rep. 2022 Feb 28;12:3339. doi: 10.1038/s41598-022-07406-x (PMC8885648; doi:10.1038/s41598-022-07406-x)
Supplement: Supplementary file 1 — Supplementary Information. [file 41598_2022_7406_MOESM1_ESM.pdf]

Integrated multi-analytical screening approach for reliable radiocarbon dating of ancient mortars

Giulia Ricci,[1,2*], Michele Secco,[2,3] Anna Addis,[4] Anna Pistilli,[1] Nereo Preto,[1] Gian Pietro Brogiolo,[3] Alexandra Chavarria Arnau,[3] Fabio Marzaioli,[5] Isabella Passariello,[5] Filippo Terrasi,[5] Gilberto Artioli,[1,2]

[1] Department of Geosciences, University of Padova, Via Giovanni Gradenigo 6, Padova 35131, Italy

[2] Inter-Departmental Research Centre for the Study of Cement Materials and Hydraulic Binders (CIRCe), University of Padova, Via Giovanni Gradenigo 6, Padova 35131, Italy

[3] Department of Cultural Heritage (DBC), University of Padova, Piazza Capitaniato 7, Padova 35139, Italy

[4] Bruker Italia S.r.l. Daltonics Division, Via Cluentina 26/R, Macerata 62010, Italy

[5] Centre for Isotopic Research on Cultural and Environmental Heritage (CIRCE), University of Campania "Luigi Vanvitelli", Viale Carlo III 153, San Nicola La Strada, Caserta 81020, Italy

*Corresponding author. GR: +39 333 7668344; giulia.ricci@unipd.it

Supplementary Materials

Supplementary Figure S1.

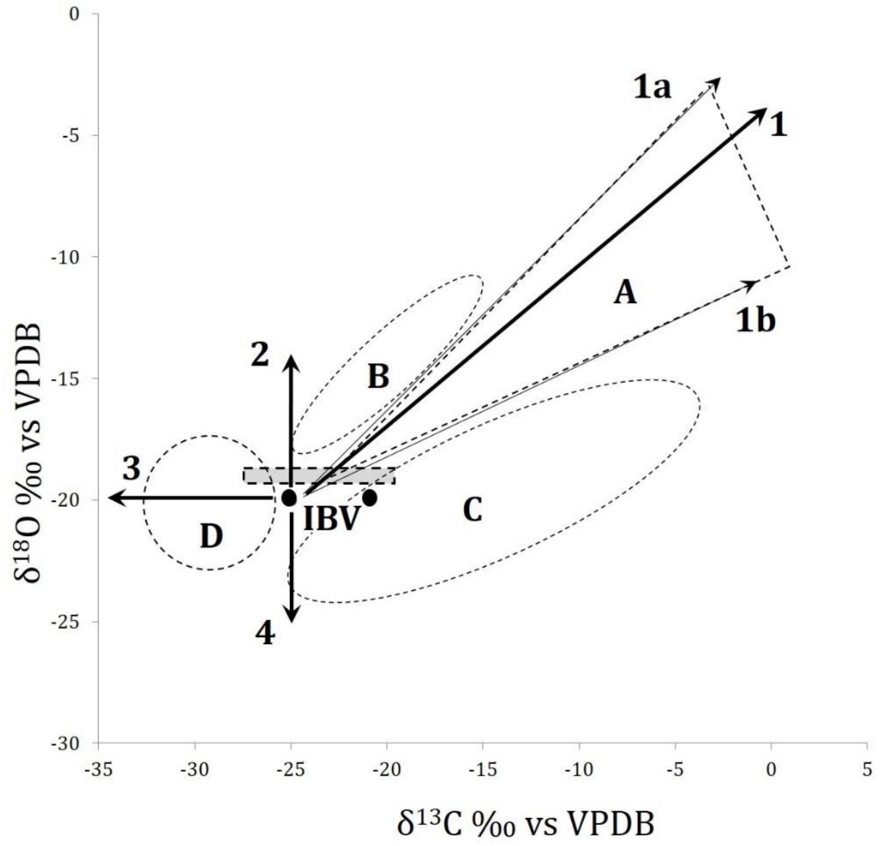

Supplementary Figure S1. Isotopic ratios ($\delta^{13}C$ and $\delta^{18}O$) diagram of a historical mortar's carbonate binder and its possible contaminations. The IBV (Ideal Binder Value) points and the grey bar represent the ideal values and range ($\delta^{13}C$ = -25 ‰ (-20.7‰) and $\delta^{18}O$ = -20 ‰; -27 ‰≤ $\delta^{13}C$≤-20 ‰ and $\delta^{18}O$ = -19 ‰) in ‰ (VPDB) as reported in literature. Following the interpretation of Kosednar-Legenstein et al. [1] area A identifies a binder containing geological limestone as contaminant. Area B indicates a shift dependent on oxygen fractionation that may be related to alteration of calcite by meteoric water, or the use of isotopically heavy water for the slaking of the putty. The isotopic values of an anthropogenic calcite subject to later dissolution and recrystallization processes or has interacted with atmospheric or biogenic CO_2 should lie in area C. The isotopic composition of anthropogenic calcite in area D is related to biogenically produced CO_2 present in the mortar preparation water or produced during biological alteration (modified from Kosednar-Legenstein et al. 2008 [1]).

Supplementary Table S1. Mineralogical quantitative phase analysis (%wt) of the bulk mortar samples obtained by XRPD according to the Rietveld method.

Sample code	Cal	Arg	Dol	LDH	Gp	Qtz	Ab	Mc	An	Orth	Ms	Chl	Am	Lz	Amph
**LOM_1**	29.4	0.0	0.0	0.0	0.0	19.6	4.4	0.0	5.3	3.8	1.9	1.2	2.3	0.0	32.1
**LOM_2**	21.9	0.0	0.0	0.0	0.0	23.2	6.4	0.0	6.0	3.8	2.1	2.0	2.6	0.0	32.2
**LOM_3**	3.6	0.0	0.1	0.3	0.0	42.3	11.4	0.0	5.1	5.7	0.0	0.1	0.0	7.3	24.1
**LOM_4**	4.1	0.0	0.0	0.0	0.0	48.4	13.8	0.0	7.1	6.8	2.0	0.3	0.0	7.9	9.6
**LOM_5**	13.4	0.0	0.0	0.0	0.0	37.2	9.2	0.0	9.7	6.5	3.3	0.4	0.0	5.0	15.4
**LOM_6**	11.5	0.0	0.0	0.6	0.0	47.2	10.1	0.0	11.8	9.8	2.1	0.2	0.0	6.3	0.3
**LOM_7**	8.9	0.0	0.0	0.0	0.0	47.0	9.9	0.0	13.1	7.2	0.9	0.3	0.0	5.0	7.7
**TOR_1**	24.5	0.0	1.6	0.0	1.1	31.9	4.1	5.6	0.0	0.0	3.4	1.4	0.2	0.0	26.3
**TOR_2**	17.2	0.0	2.4	0.9	0.0	37.9	10.9	8.5	0.0	0.0	3.2	2.1	1.7	0.0	15.2
**TOR_4**	19.1	0.0	0.6	0.0	0.0	51.3	8.0	7.0	0.0	0.0	3.3	2.2	1.6	0.0	6.9
**TOR_5**	33.3	0.0	1.4	0.0	0.2	35.8	10.0	6.5	0.0	0.0	3.3	1.2	1.3	0.0	7.0
**TOR_6**	8.5	0.0	1.1	0.0	0.0	53.0	11.9	6.1	0.0	0.0	5.1	2.9	2.4	0.3	8.8
**TOR_7**	18.3	0.0	0.5	0.0	0.0	45.8	8.2	8.0	0.0	0.0	3.1	3.6	2.5	0.0	10.0
**TOR_9**	21.5	0.0	3.3	0.4	0.0	39.7	8.8	7.0	0.0	0.0	2.8	3.0	2.2	0.0	11.5
**TOR_10**	10.7	0.0	0.3	0.0	0.0	40.9	8.5	6.9	0.0	0.0	4.3	4.2	1.6	0.0	22.5
**TOR_12**	9.0	5.0	0.5	0.0	0.0	42.1	11.0	6.5	0.0	0.0	4.2	5.9	2.4	0.0	13.4
**TOR_13**	10.0	0.0	0.2	0.0	0.0	37.8	8.0	6.7	0.0	0.0	3.3	4.5	1.1	0.0	28.6
**TOR_14**	18.9	0.0	1.1	0.6	0.0	37.6	9.2	5.4	0.0	0.0	3.9	4.5	2.2	0.0	16.7
**TOR_15**	9.6	0.0	0.0	0.0	0.0	50.4	5.1	6.1	0.0	0.0	3.9	3.2	1.4	0.0	20.3
**TOR_16**	17.2	0.0	0.4	0.0	0.0	47.6	8.1	6.7	0.0	0.0	3.1	3.1	1.6	0.0	12.2
**TOR_17**	19.1	0.0	0.9	0.0	0.0	34.1	13.3	11.5	0.0	0.0	2.9	3.8	1.5	0.0	13.0
**TOR_18**	33.3	2.8	0.5	0.0	0.0	30.3	8.4	6.2	0.0	0.0	2.4	3.1	1.8	0.0	11.0

Cal=calcite; Arg=aragonite; Dol=dolomite; LDH=layer double hydroxides; Gp=gypsum; Qtz=quartz; Ab=albite; Mc=microcline; An=anorthite; Orth=orthoclase; Ms=muscovite; Chl=chlorite; Am=amphiboles; Lz=lizardite; Amph=amorphous phases.

Supplementary text

Magnesium Silicate Hydrates

The nanostructured anthropogenic magnesium silicate hydrate (M-S-H) and magnesium aluminum silicate hydrate (M-A-S-H) phyllosilicate gels, form as secondary products during the alteration of mortars and cements by sea and ground water, interaction with clay minerals, reaction in magnesia silica-based binders [2–4].

The mortar samples from Torba, TOR_13 and TOR_15, are aerial mortars characterized by a mixture mainly made of sand and clayey soil, with the addition of a small quantity of lime, chlorite and silicate phases. The amorphous content may indicate the presence of paracrystalline phases related to hydraulic reaction products as M-S-H phases. Through the binder separation procedure, it was possible to emphasize the mineralogical composition of these samples. The obtained XRPD patterns of TOR_13_SG and TOR_15_SG samples are very similar and comparable with those of M-S-H and M-A-S-H phases reported in literature [3,4]. They are characterized by small particle size, low crystallinity and broad peaks of low diffracted intensity, as reported in Supplementary Figure S2.

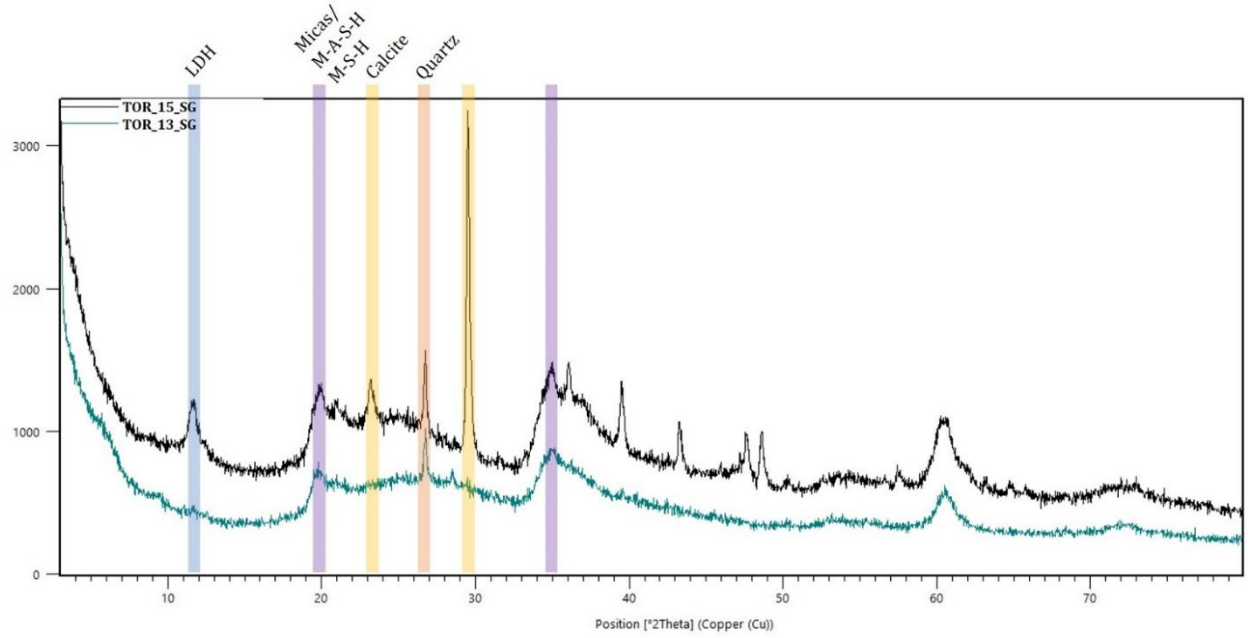

Supplementary Figure S2. X-ray diffraction (XPRD) patterns of two binder fraction samples, TOR_13_SG and TOR_15_SG characterized by the presence of magnesium aluminum silicate hydrate and magnesium silicate hydrate (M-A-S-H, M-S-H).

Supplementary Figure S3.

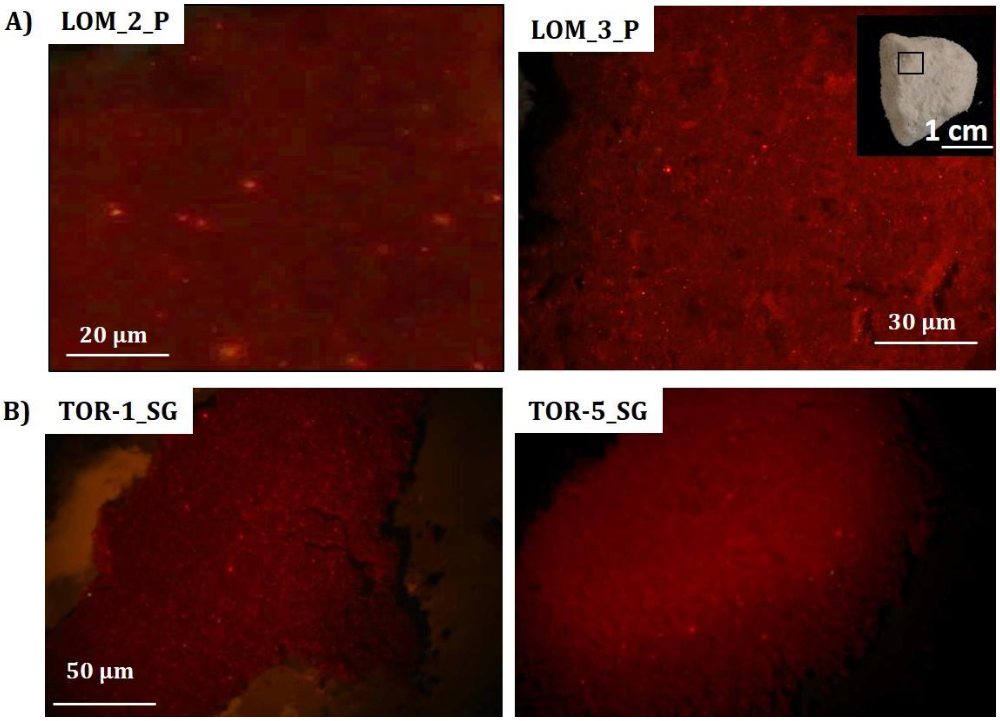

Supplementary Figure S3. Luminescence photos of representative samples. OM-CL photos of two lumps from Lomello (**A**, LOM_2_P and LOM_3_P) and two luminescence binders (SGs) from Torba (**B**, TOR_1_SG and TOR_5_SG).

References

1. Kosednar-Legenstein, B., Dietzel, M., Leis, A. & Stingl, K. Stable carbon and oxygen isotope investigation in historical lime mortar and plaster - Results from field and experimental study. *Appl. Geochemistry* **23**, 2425–2437 (2008).
2. Secco, M. *et al.* Technological transfers in the Mediterranean on the verge of Romanization: Insights from the waterproofing renders of Nora (Sardinia, Italy). *J. Cult. Herit.* **44**, (2020).
3. Bernard, E. Magnesium silicate hydrate (M-S-H) characterization: temperature, calcium, aluminum and alkali. *Phd thesis* (Université Bourgogne Franche-Comté, 2017).
4. Roosz, C. *et al.* Crystal structure of magnesium silicate hydrates (M-S-H): The relation with 2:1 Mg-Si phyllosilicates. *Cem. Concr. Res.* **73**, 228–237 (2015).
